# Idiopathic Atrophoderma of Pasini and Pierini: A Case Report and Literature Review

**DOI:** 10.7759/cureus.26571

**Published:** 2022-07-05

**Authors:** Rawan Hubail, Noor Karaidi, Ameen Al Awadhi

**Affiliations:** 1 Dermatology, Salmaniya Medical Complex, Manama, BHR

**Keywords:** dermal atrophy, iapp, morphea, pasini and pierini, atrophoderma

## Abstract

Atrophoderma of Pasini and Pierini (APP) is a rare cutaneous condition of unknown etiology, predominantly affecting young women. It is a dermatologic disorder characterized by hyperpigmented depressed patches of dermal atrophy. Patients usually present with single or multiple asymptomatic, violaceous, and sharply demarcated areas of depressed plaques; of note, unlike morphea, there is no surrounding erythema or induration. In this report, we discuss a case of a 43-year-old South Asian male patient who presented with an eight-month history of multiple asymptomatic, and depressed bluish-brown plaques that had appeared over his body. The clinicopathological correlation was consistent with a diagnosis of idiopathic atrophoderma of Pasini and Pierini (IAPP). We also engage in a review of the literature on IAPP.

## Introduction

Atrophoderma of Pasini and Pierini (APP) is an extremely uncommon dermatologic condition, with only around 200 cases reported in the medical literature so far. Idiopathic atrophoderma was first reported by Pasini in 1923, where he described the findings related to this condition as progressive atrophic dermal lesions over the trunk. Later, in 1936, Pierini and Vivoli conducted further studies on this cutaneous condition, defining and describing the lesions in more detail, as well as shedding further light on the potential link between this condition and morphea [1]. They also reported a higher prevalence of this disorder in young females. Finally, in 1958, Canizares et al. rephrased the term for this cutaneous disorder as “idiopathic atrophoderma of Pasini and Pierini (IAPP)” [2].

This dermal manifestation is usually benign in nature, presenting as sharply demarcated atrophic hyperpigmented lesions, violaceous brown in color, multiple, and varying in size with a bilateral symmetrical distribution. These lesions may also present as hypopigmented lesions or skin-colored lesions in people of color [3]. These atrophic plaques have been described previously in the literature as areas of depression in the skin mimicking “footprints in the snow” or “moth-eaten areas” with borders adopting a “cliff drop” appearance [1,4].

## Case presentation

A 43-year-old South Asian male, with no known past medical history, presented with an eight-month history of asymptomatic, hyperpigmented patches, characterized by discrete central atrophy. The lesions were violaceous in color, and ovoid in shape, appearing gradually over the dorsal aspect of hands bilaterally, and over the medial aspect of both thighs. Small multiple unilateral lesions were also noted over the lateral aspect of the right lower limb. There was no history of any recent or distant trauma, tick bites, new medication use, or any recent fungal infections reported by the patient. The patient reported that these lesions had shown the same form, texture, and color since their initial appearance, with slow and nominal growth in size noted over the course of the past eight months. Both his prior medical and familial history was deemed unremarkable. With the exception of these dermal lesions, the patient’s general health and overall physical condition were exceptional.

**Figure 1:**
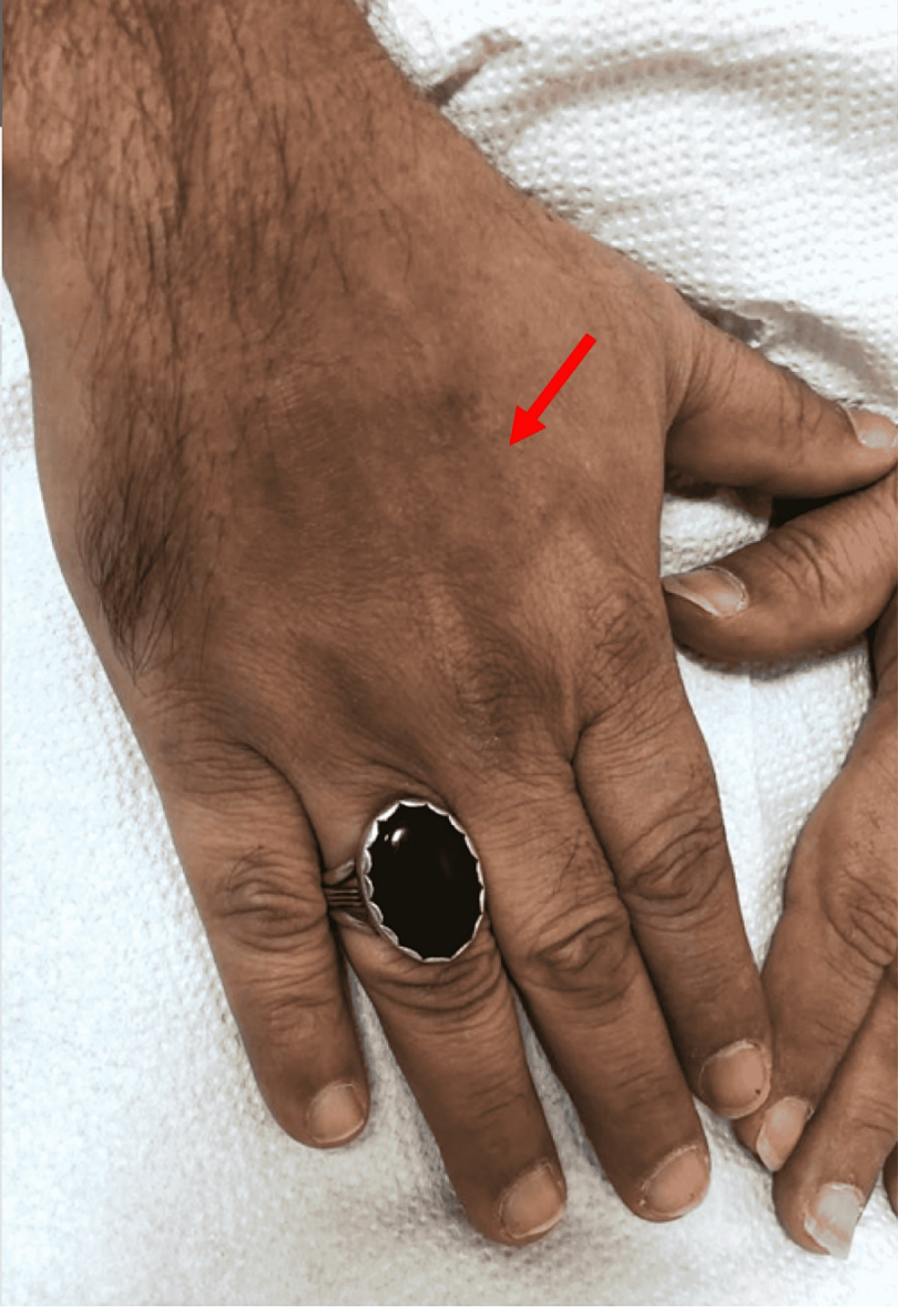
Cutaneous patch of dermal atrophy on the dorsal aspect of the right hand with a pathognomonic “cliff drop”-like appearance (arrow)

**Figure 2:**
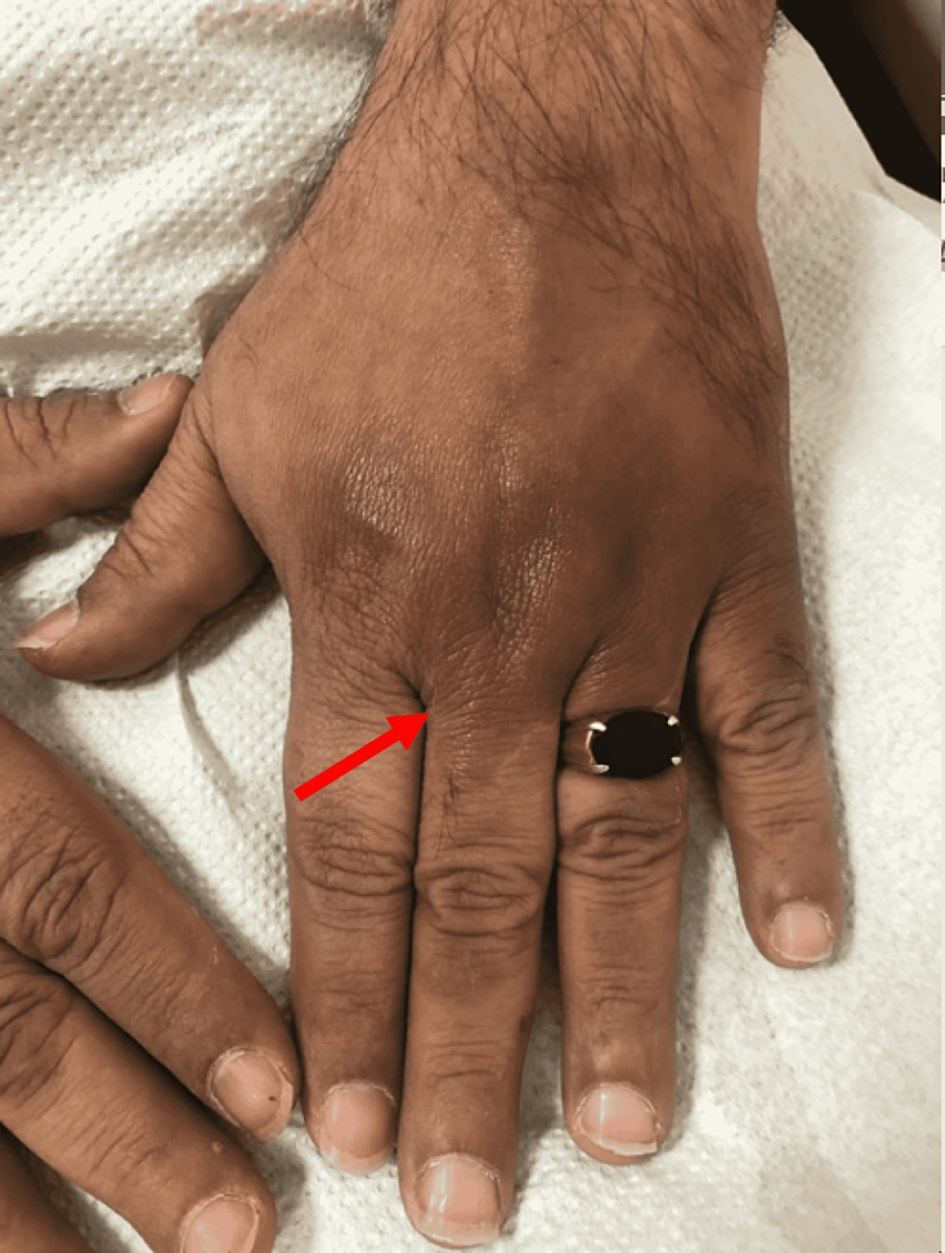
Depressed hyperpigmented violaceous cutaneous patch on the dorsal aspect of the left hand with a “cigarette paper”-like appearance (arrow)

**Figure 3:**
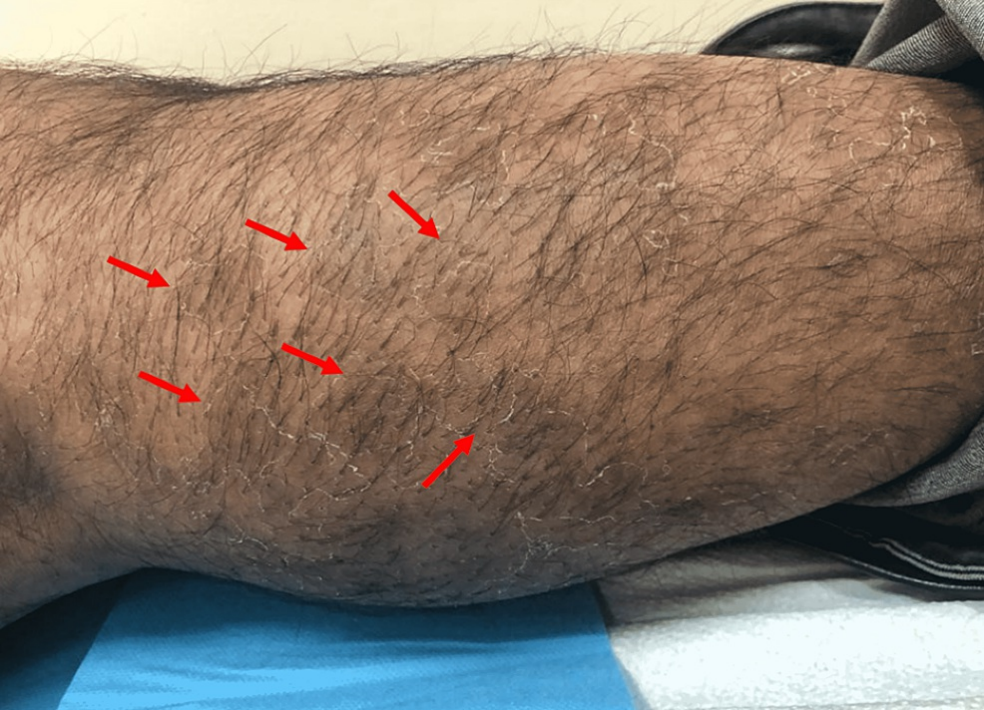
Small clustered hyperpigmented patches of dermal atrophy with a “moth-eaten” appearance on the lateral aspect of the right lower limb associated with minimal peripheral desquamation (arrows)

**Figure 4:**
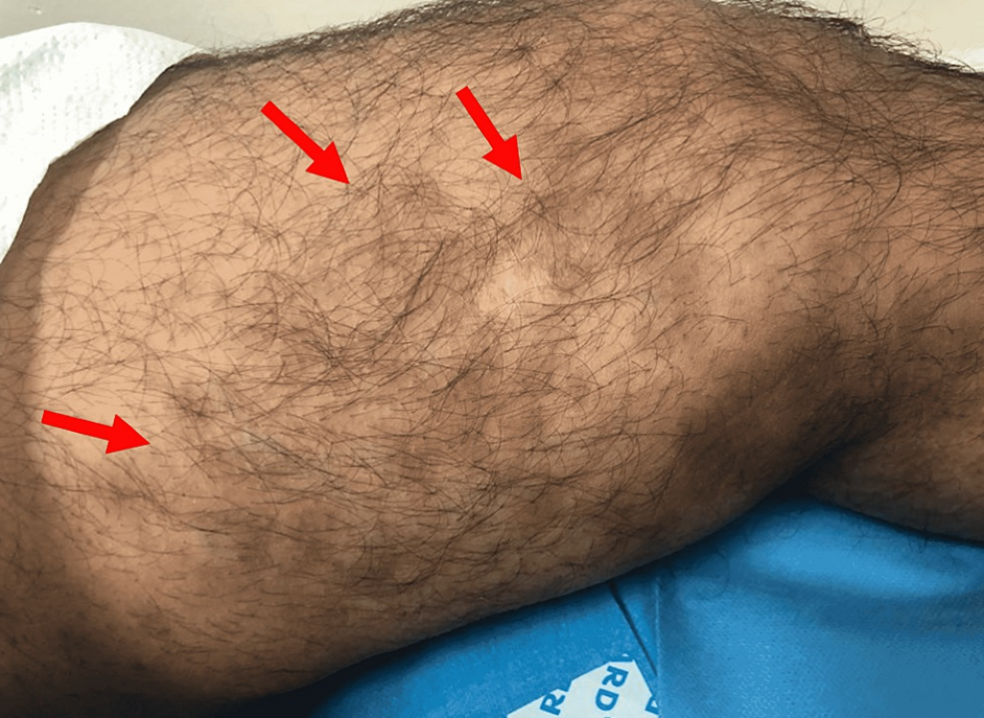
Multiple ovoid depressed cutaneous patches, brown in color and varying in size, noted on the medial aspect of the left thigh (arrows)

On dermatological examination, there were two large hyperpigmented violaceous patches with atrophic changes noted on the bilateral dorsum of hands, measuring 3 x 4 cm and 4 x 6 cm in size (Figures 1, 2). Small, multiple, brownish, and hyperpigmented patches with a depressed shiny center were noted over the lateral aspect of the right lower limb (Figure 3) as well as on the medial aspect of both thighs (Figure 4). All patches were observed to be ovoid in shape, with a central atrophic, “cigarette paper”-like appearance, conserved pathognomonic “cliff-drop” borders, and minimal surrounding peripheral desquamation. The patient reported a lightly diminished sensation within the patches, and hence a pinprick examination was performed in and around the lesions. The sensation was deemed to be intact in all areas. A list of differential diagnoses for these lesions was created based on the clinical manifestations noted, which included morphea, IAPP, leprosy, tinea corporis, psoriasis, mycosis fungoids, and pityriasis rosea.

**Figure 5:**
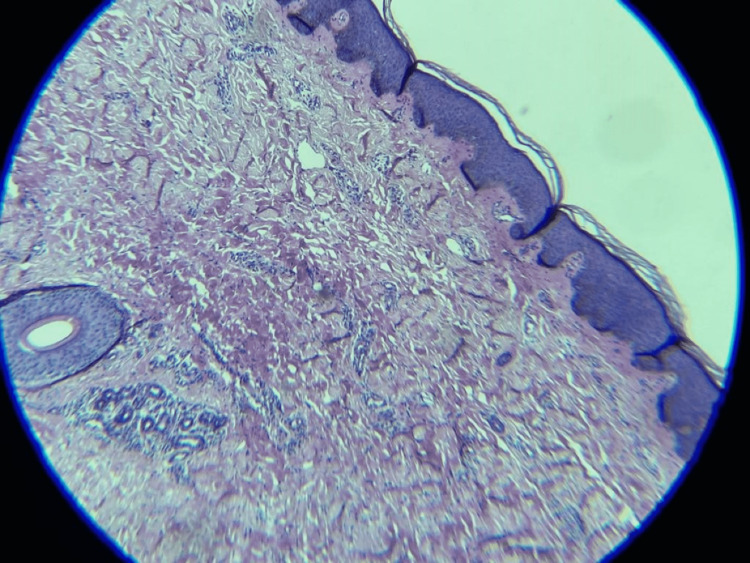
Histopathologic result of a punch biopsy taken from an atrophic plaque showing features of dermal atrophy with a hair follicle and sweat glands that are located in the reticular dermis

In order to differentiate between the various differential diagnoses, two punch biopsies were obtained, with a clean border of adjacent normal skin for further microscopic examinations and correlation (Figure 5). One biopsy was obtained from an atrophic lesion and another from the lateral surface of the right lower leg was taken to rule out fungal infection because this was the only asymmetrical and peripheral scaly lesion to be considered a fungal infection. Other lesions were bilateral symmetrical and asymptomatic. Histopathologic findings of this specimen revealed focal basal hyperpigmentation. The dermis displayed marked dermal atrophy, with mildly thickened collagen fibers and minor perivascular and periadnexal lymphocytic infiltrate. There was no obvious dermal sclerosis appreciated nor any absence of the dermal appendages. There were no signs of epidermotropism or any evidence of epidermal infiltration. The rest of the skin adnexa was unremarkable. Periodic acid-Schiff (PAS) stains were negative for fungal elements. A complete laboratory workup, including complete blood count, renal and liver function testing, serum fasting glucose test, and a lipid profile were all within normal parameters. The systemic review was also unremarkable.

After an extensive study of the clinicopathological evidence for this case, the findings were deemed consistent with the clinical impression of APP. The final diagnosis was explained to the patient and he was reassured regarding the benign course of this condition. It was also explained to him that currently there are no definitive treatments for this disorder and that the areas of depressed skin tend to be persistent, but the hyperpigmentation could be alleviated with pigment-reducing lasers, and he was advised to do so in a laser clinic.

## Discussion

IAPP is an extremely rare skin disorder. Fortunately, it is a cutaneous-limited disease with no known systemic implications. This dermal disorder has an insidious onset, and it is most commonly seen in the second and third decades of life, with a higher prevalence in women than men by a ratio of 6:1 in adults [5]. Nevertheless, APP has been reportedly seen in children below 13 years of age [6]. These atrophic patches present as asymptomatic, ovoid, slightly erythematous, and hyperpigmented violaceous patches, ranging from 1 to 20 cm in diameter, mainly over the trunk [7]. They are most commonly seen on the back and lumbosacral region [6,8]. Patches over the extremities and abdomen have also been cited in the literature but are described to be less frequent in occurrence. These lesions may present as single or multiple patches, with new discrete lesions appearing over the course of 10-20 years [9]. The diagnosis of this cutaneous condition is mainly based on clinical findings, with dermal biopsies being utilized as confirmatory tools to establish a final diagnosis. The use of ultrasonography in the differentiation between APP and other differential diagnoses has also been highlighted in the literature, whereby a decrease in dermal thickness and increase in vascularity within the atrophic plaques may be appreciated in ultrasound and Doppler imaging findings when histopathological findings are deemed to be inconclusive.

The exact etiology of this cutaneous atrophy is still unknown. The hypothesis of whether atrophoderma is a solitary entity or an atrophic variant of morphea is still being debated. APP is thought to share a pathogenetic pathway with classic morphea [10]. Indeed, atrophic lesions of “burn-out” morphea are similar to that of IAPP both from a clinical and histopathological point of view [11]. Some histological similarities such as collagen homogenization and mild sclerosis have also been noted. However, whether or not IAPP is a form of morphea is still unclear. Other clinical entities that adopt a similar form as the lesions seen in IAPP include anetoderma, long-standing lupus profundus, and atrophoderma of Moulin among others [12].

In some cases, genetic influences have also been observed to play a role in triggering APP. Studies have shown that families with a predisposition to C2 deficiency (C2D) have a higher likelihood of developing IAPP; moreover, there have been other studies that showed clustering among some families [1]. Having said that, a genetic link has not yet been confirmed. Neurogenic causes have been reported to be another possible contributing factor in certain cases of IAPP that present with a zosteriform distribution; however, evidence regarding this is lacking [13].

In another study [7], it has been reported that the results of direct immunofluorescence testing showed IgM and C3 depositions in the walls of small blood vessels within the papillary dermis, as well as focal fibrinogen found within the mid-dermis along with minimal dispersing of IgM cytoids appreciated within the basement membrane. In an electron microscopic study, results showed macrophages and lymphocytes surrounding the vessels. Yet, the relevance of these results and their significance in the diagnosis of this cutaneous disorder have not yet been elucidated [7,14]. All these findings suggest that there may be an immunological aspect to the pathogenesis of IAPP but the possible relationship is yet to be confirmed. In addition, some authors have suggested that infection caused by *Borrelia burgdorferi (B. burgdorferi)* spirochete is a trigger for this condition in some cases [9,15].

As for treatment options, there are no definitive treatments for IAPP yet. Some therapies that have been studied in the literature include hydroxychloroquine 400 mg daily for a year. Some studies suggest that it may be an effective therapeutic option for chronic refractory IAPP [16]. Hydroxychloroquine and methotrexate have also been reported to be coincidentally effective in rare cases of IAPP with associated cutaneous disorders such as lupus erythematosus or psoriasis [17]. Alternatively, oral antibiotics such as penicillin 2 million IU per day or tetracycline 500 mg three times daily for two to three weeks have shown evident improvement in patients infected with *B. burgdorferi*, and patients did not develop any new lesions during the follow-up periods ranging from three months to eight years [4,9,15]. It has also been suggested in the literature that topical corticosteroids or topical calcineurin inhibitors may also be administered as a form of milder treatment options and may exhibit positive results in the long term if an inflammatory stage of the disease is present and confirmed [15]. Looking at traditional Chinese remedies, Danshen Pill is a traditional Chinese medicine that is prescribed to patients with IAPP, and its pharmacological mechanism mainly works by promoting adequate blood circulation, dissipating blood stasis, which in turn may help with the gradual fading of these atrophic dermal patches. This form of traditional treatment has been administered in a few isolated cases but there is no proper scientific evidence to support its effectiveness in the treatment of IAPP [18]. All these treatment modalities have been reported in select cases to be successful with good long-term results; however, randomized trials on these methods are lacking and no treatment has yet been proven to effectively improve the clinical manifestations of this disease.

Given the lack of reliably effective treatment for IAPP, a conservative approach to management is preferred. In recent studies, the use of cosmetic camouflage makeup and Q-switched Alexandrite lasers have been studied in small lightly hyperpigmented lesions of IAPP to improve its cosmetic appearance and the quality of life for patients [9,19]. In a study analyzing the efficiency of Q-switched Alexandrite lasers, treated areas exhibited a 19% reduction in melanin granule size and number as well as a 65% reduction in melanosome size, volume, and number after three sessions of treatment when compared with untreated areas [20].

## Conclusions

IAPP is an uncommon cutaneous condition characterized by hyperpigmented atrophic patches, which have no definitive pathogenesis or etiology. They are brownish-violaceous lesions that appear and increase in size and number gradually and slowly over the course of many years. The relationship between morphea and IAPP is controversial and is still undergoing further study. There are no definitive treatments for this condition, but further studies are underway and appear to be promising. Emphasizing the benign nature of the disease to the patient is the most important and often the only step in the management of this condition.

## References

[1] Pullara TJ, Lober CW, Fenske NA (1984). Idiopathic atrophoderma of Pasini and Pierini. Int J Dermatol.

[2] Canizares O, Sachs PM, Jaimovich L, Torres VM (1958). Idiopathic atrophoderma of Pasini and Pierini. AMA Arch Derm.

[3] Saleh Z, Abbas O, Dahdah MJ, Kibbi AG, Zaynoun S, Ghosn S (2008). Atrophoderma of Pasini and Pierini: a clinical and histopathological study. J Cutan Pathol.

[4] Buechner SA, Rufli T (1994). Atrophoderma of Pasini and Pierini: clinical and histopathologic findings and antibodies to Borrelia burgdorferi in thirty-four patients. J Am Acad Dermatol.

[5] Bassi A, Remaschi G, Difonzo EM (2015). Idiopathic congenital atrophoderma of Pasini and Pierini. Arch Dis Child.

[6] Soorya B, Thomas J, Manoharan D, Rajendran SN (2015). Atrophoderma of Pasini and Pierini - a case report. Biomed Pharmacol J.

[7] Calka O, Metin A, Kosem M (2001). Idiopathic atrophoderma of Pasini and Pierini. East J Med.

[8] Avancini J, Valente NY, Romiti R (2015). Generalized lenticular atrophoderma of Pasini and Pierini. Pediatr Dermatol.

[9] (2022). Atrophoderma of Pasini and Pierini. https://www.uptodate.com/contents/atrophoderma-of-pasini-and-pierini.

[10] Murphy PK, Hymes SR, Fenske NA (1990). Concomitant unilateral idiopathic atrophoderma of Pasini and Pierini (IAPP) and morphea. Observations supporting IAPP as a variant of morphea. Int J Dermatol.

[11] Kim SK, Rhee SH, Kim YC, Lee ES, Kang HY (2006). Congenital atrophoderma of Pasini and Pierini. J Korean Med Sci.

[12] Jablonska S, Blaszczyk M (2004). Is superficial morphea synonymous with atrophoderma Pasini-Pierini?. J Am Acad Dermatol.

[13] Arif T (2019). Zosteriform idiopathic atrophoderma of Pasini and Pierini. Indian J Paediatr Dermatol.

[14] Berman A, Berman GD, Winkelmann RK (1988). Atrophoderma (Pasini-Pierini). Findings on direct immunofluorescent, monoclonal antibody, and ultrastructural studies. Int J Dermatol.

[15] Lee Y, Oh Y, Ahn SY, Park HY, Choi EH (2011). A case of atrophoderma of Pasini and Pierini associated with Borrelia burgdorferi infection successfully treated with oral doxycycline. Ann Dermatol.

[16] Carter JD, Valeriano J, Vasey FB (2006). Hydroxychloroquine as a treatment for atrophoderma of Pasini and Pierini. Int J Dermatol.

[17] Eichhoff G (2019). Atrophoderma of Pasini and Pierini in a patient with concomitant psoriasis: response to methotrexate. JAAD Case Rep.

[18] Zhang RZ, Zhu WY (2011). Two uncommon cases of idiopathic atrophoderma of pasini and pierini: multiple and giant. Indian J Dermatol Venereol Leprol.

[19] Salsberg JM, Weinstein M, Shear N, Lee M, Pope E (2016). Impact of cosmetic camouflage on the quality of life of children with skin disease and their families. J Cutan Med Surg.

[20] Arpey CJ, Patel DS, Stone MS, Qiang-Shao J, Moore KC (2000). Treatment of atrophoderma of Pasini and Pierini-associated hyperpigmentation with the Q-switched alexandrite laser: a clinical, histologic, and ultrastructural appraisal. Lasers Surg Med.

